# Prevalence and risk indicators of first-wave COVID-19 among oral health-care workers: A French epidemiological survey

**DOI:** 10.1371/journal.pone.0246586

**Published:** 2021-02-11

**Authors:** Sébastien Jungo, Nathan Moreau, Marco E. Mazevet, Anne-Laure Ejeil, Martin Biosse Duplan, Benjamin Salmon, Violaine Smail-Faugeron

**Affiliations:** 1 UFR Odontologie, Dental Medicine Department, AP-HP, Bretonneau Hospital, Université de Paris, Paris, France; 2 Laboratory of Orofacial Neurobiology (EA7543), Université de Paris, Paris, France; 3 Dental Innovation and Translation Hub, Faculty of Dentistry, Oral & Craniofacial Sciences, Kings College London, Guy’s Hospital, Tower Wing, London, United Kingdom; 4 Laboratory of Orofacial Pathologies, Imaging and Biotherapies UR2496, Université de Paris, Montrouge, France; 5 INSERM U1163, Imagine Institut; 6 Dental Materials Research Unit (URB2I), Université de Paris, Montrouge, France; Eberhard-Karls-Universitat Tubingen Medizinische Fakultat, GERMANY

## Abstract

**Background:**

Previous studies have highlighted the increased risk of contracting the COVID-19 for health-care workers and suggest that oral health-care workers may carry the greatest risk. Considering the transmission route of the SARS-CoV-2 infection, a similar increased risk can be hypothesized for other respiratory infections. However, no study has specifically assessed the risk of contracting COVID-19 within the dental profession.

**Methods:**

An online survey was conducted within a population of French dental professionals between April 1 and April 29, 2020. Univariable and multivariable logistic regression analyses were performed to explore risk indicators associated with laboratory-confirmed COVID-19 and COVID-19-related clinical phenotypes (i.e. phenotypes present in 15% or more of SARS-CoV-2-positive cases).

**Results:**

4172 dentists and 1868 dental assistants responded to the survey, representing approximately 10% of French oral health-care workers. The prevalence of laboratory-confirmed COVID-19 was 1.9% for dentists and 0.8% for dental assistants. Higher prevalence was found for COVID-19-related clinical phenotypes both in dentists (15.0%) and dental assistants (11.8%). Chronic kidney disease and obesity were associated with increased odds of laboratory-confirmed COVID-19, whereas working in a practice limited to endodontics was associated with decreased odds. Chronic obstructive pulmonary disease, use of public transportation and having a practice limited to periodontology were associated with increased odds of presenting a COVID-19-related clinical phenotype. Moreover, changes in work rhythm or clinical practice were associated with decreased odds of both outcomes.

**Conclusions:**

Although oral health-care professionals were surprisingly not at higher risk of COVID-19 than the general population, specific risk indicators could exist, notably among high aerosol-generating dental subspecialties such as periodontology. Considering the similarities between COVID-19-related clinical phenotypes other viral respiratory infections, lessons can be learned from the COVID-19 pandemic regarding the usefulness of equipping and protecting oral health-care workers, notably during seasonal viral outbreaks, to limit infection spread.

**Impact:**

Results from this study may provide important insights for relevant health authorities regarding the overall infection status of oral health-care workers in the current pandemic and draw attention to particular at-risk groups, as illustrated in the present study. Protecting oral health-care workers could be an interesting public health strategy to prevent the resurgence of COVID-19 and/or the emergence of new pandemics.

## Introduction

On March 13, 2020, the World Health Organization (WHO) declared that Europe had become the new epicenter of the Coronavirus Disease 2019 (COVID-19) pandemic. France was particularly affected with 29,965 deaths at the time of writing (July 9, 2020). 168,810 cases were confirmed in this country, including more than 30,000 (18%) health-care workers. This prevalence is underestimated: at this time, testing was limited by the availability of diagnostic tools [1, 2]. As they are on the front line, health-care workers have an increased risk of contracting COVID-19 [3, 4]. Several studies show that between 3.8% of health-care workers in Wuhan to 20% in Italy were infected, leading to several fatal outcomes [5, 6]. In particular, oral health-care workers could be among the most exposed health-care workers to severe acute respiratory syndrome coronavirus 2 (SARS-CoV-2) infections, because they are frequently subjected to saliva projections and aerosols [7]. To prevent the risk of cross-contamination between dental professionals and their patients, dental practices were urged to upgrade their personal protective equipment (PPE) standards following the pandemic outbreak [8]. These guidelines were country-based, with inconsistent recommendations across borders. Furthermore, these professionals did not have the required PPE due to severe shortages, increasing the spread of the infection [9]. Such issues are not specific of COVID-19 and could concern other respiratory viruses also responsible for high mortality pandemics, such as the 2009 H1N1 virus outbreak [10], since they are also easily transmitted by direct contact or airborne droplets [11]. It has been suggested that geographic expansion and genetic recombination of viruses such as H7N9 or H5N1 could constitute the next pandemic [12]. Considering their high aerosol-generating activity, notably during ultrasonic scaling and high-speed drilling with irrigation, dental practices could thus play a key role in the transmission of such respiratory viruses.

Previous studies have highlighted the increased risk of COVID-19 in health-care workers and suggest that dental professionals could carry the highest risk [13, 14]. Moreover, since June 25, 2020, the WHO has warned of the potential risk of COVID-19 resurgence in Europe. However, to the best of our knowledge, data is lacking on the risk of contracting COVID-19 or developing one of the clinical phenotypes associated with COVID-19 [15] for oral health-care workers. Such focus is of importance, both to mitigate the possible increased morbidity/mortality risk among oral professionals and to limit cross-contamination with patients. Therefore, this study aimed to survey French oral health-care workers (1) to report the prevalence of COVID-19 and COVID-19-related clinical phenotypes, (2) to describe putative exposure history, (3) to identify risk indicators associated with COVID-19 and clinical phenotypes associated with COVID-19 development, and (4) to assess the self-reported stress levels caused by the first-wave pandemic. Such data may provide valuable insights for relevant policymakers and dental organizations regarding the overall infection status of oral health-care workers, eventually leading to new health-care policies on prophylactic measures for the resurgence of COVID-19 and/or the emergence of new pandemics.

## Methods

To assess the impact of the pandemic within the French dental profession, an anonymous, non-incentivized, online survey was conducted in accordance with the 1964 Helsinki declaration and approved by the French national authorities regulating confidentiality (CNIL, Commission Nationale Informatique et Libertés, No. 2217408). Participants were informed of the data collection, study aims and relevant data protection measures.

### Survey setting and participants

From April 1 to April 29, 2020 a survey was conducted via the online software Google Forms^®^ (chosen for ease of use and hypothesized familiarity among the surveyed population), aimed at French dental practitioners and dental assistants, using a snowball sampling method [16]. The survey was disseminated via the national dental association, deans of dental faculties, scientific societies, professional social networks, and people were encouraged to pass it on to their peers. Prior to its dissemination, the survey was tested on twenty participants. They tested several scenarios (e.g., having a laboratory-confirmed COVID-19 status or not, being symptomatic or not…) and reported their comprehension difficulties regarding the questions; subsequent adaptations were made to improve their clarity. All survey questions were mandatory (i.e. required adequate completion before getting access to the next question) thus guaranteeing a lack of incomplete data in this study.

### Survey development

The structured questionnaire consisted of 78 questions divided in 23 sections, with a mean number of questions per section of four. Depending on their answers, participants did not have to complete all sections, and had on average between 27 and 37 questions to answer out of the 78 (see S1 Fig). Total completion was estimated to take less than five minutes (thus promoting precise and detailed answers). Several categories were covered: sociodemographic data; health status; work environment; perceived stress relating to the COVID-19 pandemic; COVID-19 status; and exposure history. Potential changes before and during lockdown, enforced by the French government from March 17, to May 11, 2020, were also assessed. Sociodemographic data were collected on gender, age, household size, and parental status. Health status variables included tuberculosis vaccination (BCG) status (a putative protective factor, investigated in other undergoing clinical trials: NCT04327206, NCT04328441) and the presence of several medical conditions (suspected risk factors for severe COVID-19 at the time of survey), such as allergies, diabetes, hypertension, cardiopathies, pulmonary or kidney diseases, malignancies, obesity, and immunodeficiencies [17]. Work environment characteristics included professional orientation (some dental specialties could carry higher risk than others depending on aerosol production levels) and the use of public transportation. Perceived stress levels of respondents were assessed with a numerical rating scale (NRS) ranging from 0 (no stress) to 10 (highest stress imaginable) [18], regarding their personal safety, the safety of their families, and the financial stability of their professional practice. COVID-19 status included predefined symptom inquiries (fever, chills, rhinitis, sore throat, cough, anosmia, agueusia, dyspnea, acute respiratory distress syndrome, headache, conjunctivitis, vertigo, myalgia) [19], COVID-19-related clinical phenotypes (i.e. phenotypes present in 15% or more of SARS-CoV-2-positive cases, according to Smith et al. [15]), date of first symptom appearance, and viral laboratory-testing. Symptomatic respondents (i.e. having one or more predefined symptoms) were asked to define putative exposure history, including contact history (within private or work environment) and professional exposure (i.e. clinical interview vs. dental procedures, number and age of treated patients and types of PPE) that occurred in the past 15 days before symptom onset. Finally, the proportion of children under 5 years of age treated in dental practices was specifically addressed as some authors have suggested that such children have high nasopharyngeal SARS-CoV-2 viral loads compared to older children and adults, and would thus be especially contagious [20].

### Data synthesis and analysis

Study sample data were described using frequencies (percentages) for qualitative variables and median (interquartile range (IQR), minimum-maximum (Min-Max)) for quantitative variables. When appropriate, Mann-Whitney U test or Kruskal-Wallis were used for quantitative variables and Fisher’s exact test for binary variables to compare differences between laboratory-confirmed COVID-19 positive vs. COVID-19 negative cases & non-tested practitioners, symptomatic vs. asymptomatic cases, and tested vs. non-tested cases. To explore the associated risk indicators, univariable and multivariable logistic regression analyses were performed. Variables with p value ≤ 0.2 in the univariable analysis were introduced into multivariable logistic regression analysis.

As some non-tested practitioners could be positive for COVID-19 while others tested negative could actually be infected (false negatives), the probability of being infected by COVID-19 among non-tested and false negative respondents was estimated by a predictive model using a binary logistic regression [21]. The predictive model was designed to compensate for lack of testing at the time of study design, i.e. to assess the potential underestimation of COVID-19 prevalence. Indeed, in France, SARS-CoV-2 testing was neither automatic nor generalized at the time of study design, and only specific symptomatic cases could be tested as per national health-care regulations of the time (www.santepubliquefrance.fr/maladies-et-traumatismes/maladies-et-infections-respiratoires/infection-a-coronavirus/documents/affiche/alerte-coronavirus-les-tests-de-depistage-ne-sont-pas-automatiques-affiche-a4-francais). "Laboratory-confirmed COVID-19" was used as dependent variable and age, gender, and symptoms as independent variables. Gender, fever, sore throat, tiredness, and ageusia were selected with backward stepwise selection. To build the model, the number of laboratory-confirmed COVID-19 dentists was split into two sets: training (65%) and test (35%) datasets. These datasets were selected at random. The accuracy of the model was confirmed by the cross-validation area under the receiver operating curve (ROC) equal to 0.89 for training dataset and 0.76 for test dataset. For test dataset, positive and negative predictive values were 63.6% and 72.9%, respectively, sensitivity and specificity 51.9% and 81.4%, respectively. The model was then applied to the entire symptomatic population. Participants were predicted positive for COVID-19 when the probability estimated by the model was strictly greater than 50%. A p < 0.05 was considered statistically significant. Analyses were performed using R software (version 3.6.2; https://www.r-project.org).

## Results

In total, 4172 dental practitioners and 1868 dental assistants responded to the questionnaire, which corresponds to approximately 10% of French oral health-care workers.

### Socio-demographic data, health status and clinical practice

The median age of the 4172 dentists was 44 years (IQR, 34 to 55), ranging from 21 to 86 years, and more than half were women, and had children. BCG vaccination coverage rate was high among dentists (3151 [75.5%]). Medical conditions were reported in a quarter of dentists. The most common medical condition was hypertension, followed by chronic obstructive pulmonary disease and cardiopathies. Most dentists worked in private practices (3858 [92.5%]). General practice was the most represented practice (3508 [84.1%]) in the sample, followed by practice limited to periodontology and practice limited to oral surgery. After the enforcement of nationwide lockdown, the use of public transportation was reduced by 42% (406 [9.7%] vs 170 [4.1%]). Most dentists did not report any changes in their familial environment (3063 [73.4%]), whereas they did in their working environment (4008 [96.1%]). This was due to changes in clinical practice (e.g. participation in telephone regulation of emergency cases) (2966 [71.1%]) and/or work rhythm (e.g. practice limited to emergencies only) (1412 [33.8%]). Data related to dentists are summarized in Table 1.

**Table 1:** Socio-demographic data, health status, clinical practice, changes after enforcement of lockdown and COVID status of all dental practitioners.

	All included dentists (n = 4172)	No test performed (n = 3973)	Tested Negative (n = 120)	Tested Positive (n = 79)	p-value (1)	No COVID-19-related clinical phenotypes (n = 3546)	COVID-19-related clinical phenotypes (n = 626)	p-value (2)
**Demographic data**								
Age, years	44.00 [34.00, 55.00]	44.00 [34.00, 55.00]	43.00 [35.00, 54.00]	44.00 [36.00, 53.00]	0.515*	**44.00 [35.00, 55.75]**	**41.00 [34.00, 52.00]**	**<0.001**^**#**^
Male gender	1791 (42.9)	1710 (43.0)	44 (36.7)	37 (46.8)	0.296	**1560 (44.0)**	**231 (36.9)**	**0.001**
≥ 1 child	1853 (44.4)	44 (36.7)	1780 (44.8)	29 (36.7)	0.08	1580 (44.6)	273 (43.6)	0.692
**Medical Conditions**								
Current pregancy	79 (1.9)	75 (1.9)	2 (1.7)	2 (2.5)	0.902	68 (1.9)	11 (1.8)	0.91
Current Smoking	372 (8.9)	358 (9.0)	7 (5.8)	7 (8.9)	0.485	314 (8.9)	58 (9.3)	0.798
Comorbidities								
Allergies	31 (0.7)	27 (0.7)	2 (1.7)	2 (2.5)	0.081	24 (0.7)	7 (1.1)	0.351
Diabetes	70 (1.7)	69 (1.7)	0 (0.0)	1 (1.3)	0.331	62 (1.7)	8 (1.3)	0.499
Hypertension	270 (6.5)	255 (6.4)	7 (5.8)	8 (10.1)	0.398	233 (6.6)	37 (5.9)	0.595
Cardiopathies	120 (2.9)	114 (2.9)	4 (3.3)	2 (2.5)	0.94	104 (2.9)	16 (2.6)	0.696
COPD	156 (3.7)	145 (3.6)	9 (7.5)	2 (2.5)	0.077	**122 (3.4)**	**34 (5.4)**	**0.021**
CKD	18 (0.4)	**15 (0.4)**	**1 (0.8)**	**2 (2.5)**	**0.012**	14 (0.4)	4 (0.6)	0.597
Malignancies	93 (2.2)	91 (2.3)	1 (0.8)	1 (1.3)	0.478	84 (2.4)	9 (1.4)	0.191
Obesity	97 (2.3)	**88 (2.2)**	**4 (3.3)**	**5 (6.3)**	**0.042**	81 (2.3)	16 (2.6)	0.786
ID	47 (1.1)	45 (1.1)	1 (0.8)	1 (1.3)	0.948	37 (1.0)	10 (1.6)	0.315
Other	140 (3.4)	133 (3.3)	4 (3.3)	3 (3.8)	0.976	119 (3.4)	21 (3.4)	1
**Clinical practice**								
Specialty								
General practice	3508 (84.1)	3352 (84.4)	93 (77.5)	63 (79.7)	0.073	**3005 (84.7)**	**503 (80.4)**	**0.007**
Endodontics	397 (9.5)	383 (9.6)	12 (10.0)	2 (2.5)	0.101	343 (9.7)	54 (8.6)	0.454
Oral surgery	636 (15.2)	599 (15.1)	23 (19.2)	14 (17.7)	0.389	529 (14.9)	107 (17.1)	0.182
Orthodontics	414 (9.9)	400 (10.1)	10 (8.3)	4 (5.1)	0.284	353 (10.0)	61 (9.7)	0.928
Pediatric dentistry	294 (7.0)	279 (7.0)	12 (10.0)	3 (3.8)	0.238	**236 (6.7)**	**58 (9.3)**	**0.023**
Restorative dentistery	369 (8.8)	353 (8.9)	14 (11.7)	2 (2.5)	0.078	317 (8.9)	52 (8.3)	0.662
Periodontology	644 (15.4)	**605 (15.2)**	**28 (23.3)**	**11 (13.9)**	**0.05**	**524 (14.8)**	**120 (19.2)**	**0.006**
Prosthodontics	610 (14.6)	579 (14.6)	21 (17.5)	10 (12.7)	0.592	517 (14.6)	93 (14.9)	0.905
Implantology	139 (3.3)	128 (3.2)	6 (5.0)	5 (6.3)	0.184	112 (3.2)	27 (4.3)	0.173
Disability	6 (0.1)	6 (0.2)	0 (0.0)	0 (0.0)	0.86	4 (0.1)	2 (0.3)	0.493
Gnathology	82 (2.0)	76 (1.9)	3 (2.5)	3 (3.8)	0.447	71 (2.0)	11 (1.8)	0.802
Other	27 (0.6)	**24 (0.6)**	**3 (2.5)**	**0 (0.0)**	**0.03**	**17 (0.5)**	**10 (1.6)**	**0.003**
Private practice	3858 (92.5)	3680 (92.6)	110 (91.7)	68 (86.1)	0.087	3290 (92.8)	568 (90.7)	0.088
Working in group practice	574 (13.8)	536 (13.5)	21 (17.5)	17 (21.5)	0.059	478 (13.5)	96 (15.3)	0.238
Number of staff								
Medical	2.00 [2.00, 4.00]	**2.00 [2.00, 4.00]**	**2.50 [2.00, 4.00]**	**3.00 [2.00, 5.50]**	0.008^*^	**2.00 [2.00, 4.00]**	**3.00 [2.00, 4.00]**	**0.03**^**#**^
Non-medical	2.00 [1.00, 4.00]	**2.00 [1.00, 4.00]**	**3.00 [2.00, 5.25]**	**4.00 [2.00, 7.00]**	0.005^*^	**2.00 [1.00, 4.00]**	**3.00 [2.00, 5.00]**	**0.002**^**#**^
Taking public transportation	457 (11.0)	**425 (10.7)**	**18 (15.0)**	**14 (17.7)**	**0.05**	**360 (10.2)**	**97 (15.5)**	**<0.001**
Before lockdown	406 (9.7)	**377 (9.5)**	**15 (12.5)**	**14 (17.7)**	**0.029**	**321 (9.1)**	**85 (13.6)**	**0.001**
After lockdown	170 (4.1)	160 (4.0)	7 (5.8)	3 (3.8)	0.61	**132 (3.7)**	**38 (6.1)**	**0.009**
**Changes after lockdown**								
Family environement								
Household size increase	689 (16.5)	664 (16.7)	16 (13.3)	9 (11.4)	0.287	**611 (17.2)**	**78 (12.5)**	**0.004**
Household size decrease	255 (6.1)	**236 (5.9)**	**14 (11.7)**	**5 (6.3)**	**0.036**	206 (5.8)	49 (7.8)	0.064
Relocation	172 (4.1)	166 (4.2)	4 (3.3)	2 (2.5)	0.696	**136 (3.8)**	**36 (5.8)**	**0.035**
Other	33 (0.8)	31 (0.8)	0 (0.0)	2 (2.5)	0.134	**23 (0.6)**	**10 (1.6)**	**0.026**
Work environement								
No change	284 (6.8)	**268 (6.7)**	**5 (4.2)**	**11 (13.9)**	**0.022**	**224 (6.3)**	**60 (9.6)**	**0.004**
Workplace	7 (0.2)	7 (0.2)	0 (0.0)	0 (0.0)	0.839	7 (0.2)	0 (0.0)	0.56
Work rhythm	1412 (33.8)	1353 (34.1)	39 (32.5)	20 (25.3)	0.254	**1264 (35.6)**	**148 (23.6)**	**<0.001**
Clinical practice	2966 (71.1)	**2852 (71.8)**	**78 (65.0)**	**36 (45.6)**	**<0.001**	**2556 (72.1)**	**410 (65.5)**	**0.001**
Reduce number of medical staff	794 (19.0)	**767 (19.3)**	**12 (10.0)**	**15 (19.0)**	**0.038**	686 (19.3)	108 (17.3)	0.24
Reduce number of paramedical staff	1420 (34.0)	**1368 (34.4)**	**27 (22.5)**	**25 (31.6)**	**0.022**	**1242 (35.0)**	**178 (28.4)**	**0.002**
Reduce number of administrative staff	898 (21.5)	855 (21.5)	23 (19.2)	20 (25.3)	0.586	778 (21.9)	120 (19.2)	0.133
Work stopping	136 (3.3)	**120 (3.0)**	**9 (7.5)**	**7 (8.9)**	**<0.001**	**103 (2.9)**	**33 (5.3)**	**0.003**
**COVID-19 status**								
COVID-19-related clinical phenotypes	626 (15.0)	**501 (12.6)**	**57 (47.5)**	**68 (86.1)**	**<0.001**			
Date of first symptoms	2020-03-14 [2020-03-05, 2020-03-20]	**2020-03-14 [2020-03-03, 2020-03-19]**	**2020-03-18 [2020-03-13, 2020-03-21]**	**2020-03-17 [2020-03-13, 2020-03-21]**	<0.001^*^	**2020-03-15 [2020-03-07, 2020-03-20]**	**2020-03-14 [2020-03-04, 2020-03-19]**	**0.024**^**#**^
Test								**<0.001**
None	3973 (95.2)					**3472 (97.9)**	**501 (80.0)**	
Negative	120 (2.9)					**63 (1.8)**	**57 (9.1)**	
Positive	79 (1.9)					**11 (0.3)**	**68 (10.9)**	

Data are median [IQR], n (%). P-values comparing (1) dentists’ COVID-19 test status (no test, negative or positive) and (2) dentists with a COVID-19-related clinical phenotype vs not, are from (#) Mann-Whitney U test, (*) Kruskal-Wallis or Fisher’s exact test when not specified. COPD: chronic obstructive pulmonary disease; CKD: chronic kidney disease; ID: immunodeficiencies; Lockdown was enforced from March 17 to May 11, 2020.

Compared to dentists, dental assistants were younger (38 vs 44 years), with a larger proportion of females (98.2% vs 57.1%) and a higher prevalence of obesity (4.6% vs 2.3%). They were less often represented in general practice (80.3% vs 84.1%) and more often in practices limited to periodontology (31.5% vs 15.4%) and pediatric dentistry (8.9% vs 7.0%). Compared to dentists, dental assistants used public transportation more often (17.0% vs 11.0%), either before or during lockdown, did not change their familial environment as often (82.7% vs 73.4%) and most stopped their professional activity during lockdown (77.7% vs 3.3%). Data related to dental assistants are given in S1 Table.

### Prevalence of COVID-19 and COVID-19-related clinical phenotypes

#### COVID-19

The prevalence of laboratory-confirmed COVID-19 was 1.9% (n = 79) for dentists and 0.8% (n = 14) for dental assistants. However, only 199 (4.8%) dentists and 36 (1.9%) dental assistants were laboratory-tested. When applying the predictive model, prevalences were increased by a factor of 2.5 for dentists (5%, n = 207) and by a factor of 3 for dental assistants (2.5%, n = 46). Fewer dental assistants were tested than dentists, while the proportion of laboratory-confirmed COVID-19 cases was similar in both groups (38.9% vs 39.7%). Overall, respondents who were tested had particular profiles. For example, tested dentists were more often symptomatic than those not tested (see S2 Table), and living with children, having allergies or being obese were associated with increased odds of being tested (see S3 Table).

#### COVID-19-related clinical phenotypes

Clinical phenotypes associated with COVID-19 were explored to account for the variability of COVID-19 testing accuracy and availability. The prevalence of COVID-19-related clinical phenotypes was 15.0% (n = 626) for dentists and 11.8% (n = 220) for dental assistants. The different clinical phenotypes associated with COVID-19 are detailed in S4 Table.

### Putative exposure history

**Fig 1:**
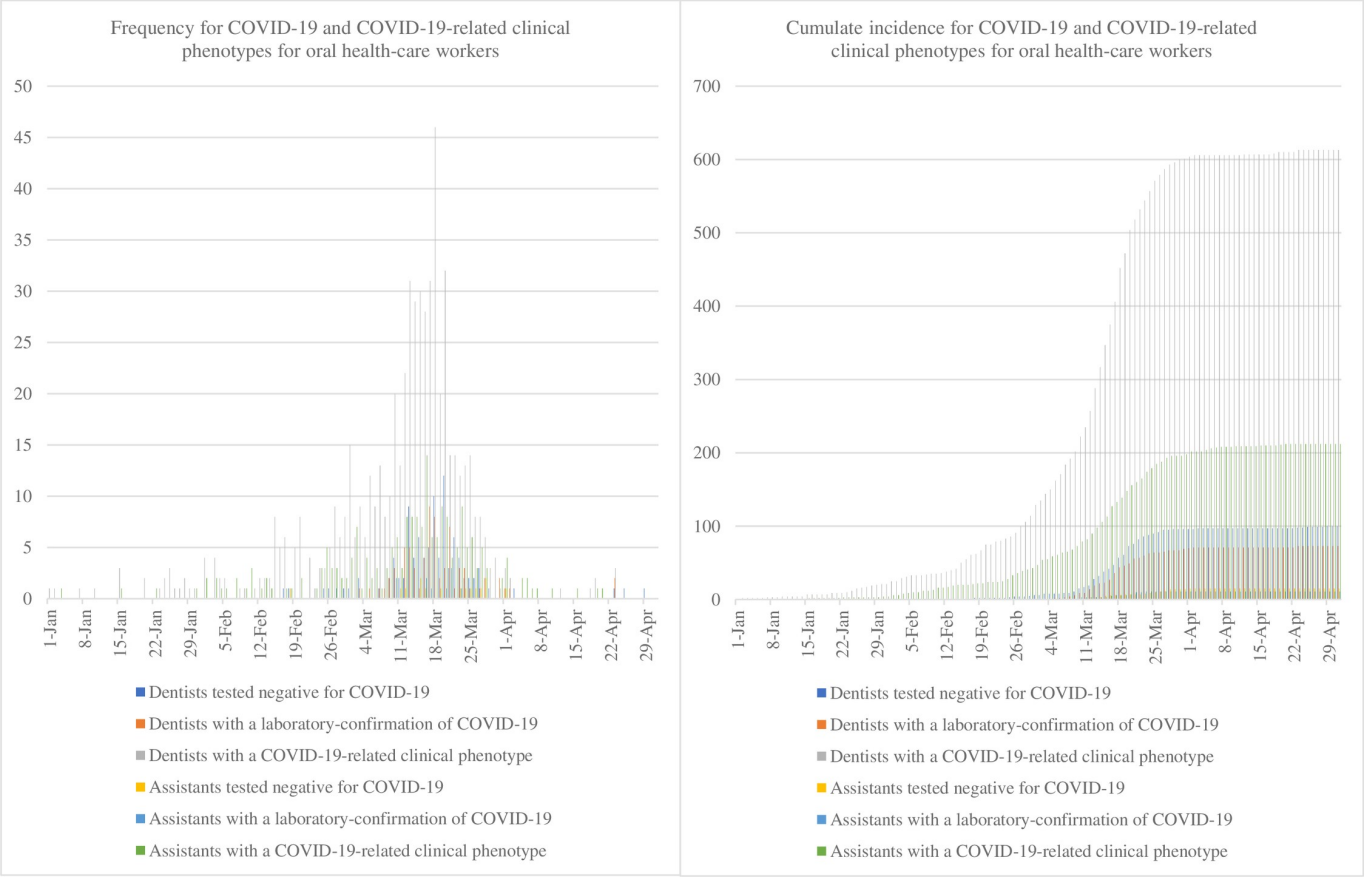
Epidemic curves for COVID-19 and COVID-19-related clinical phenotypes for oral health-care workers in France, 2020: (a) frequency and (b) cumulative incidence.

Data regarding putative exposure history are given in Table 2. Among symptomatic respondents, 373 (45.9%) suspected a transmission within their work environment, whereas only 130 (11.9%) suspected a transmission within the private sphere. Almost half of dentists had performed dental procedures in the 15 days preceding the onset of symptoms and three-quarters had treated patients with no specific measures (only gloves and surgical mask). Comparatively, dental assistants used FFP2 masks (3.9% vs 8.8%), safety googles (39.2% vs 62.0%) and hairnets (7.2% vs 12.1%) less frequently (see S5 Table). The median date of symptom apparition was March 14, 2020 (IQR, March 5 to 20) for dentists with laboratory-confirmed COVID-19 and March 15, 2020 (IQR, March 4 to 22) for dental assistants. A sharp increase of dentists with COVID-19-related clinical phenotypes was seen around February 22, 2020 leading to a peak around March 18, 2020, when 46 cases were reported (Fig 1A). This distribution of daily number of symptomatic cases was different from that of laboratory-confirmed COVID-19 cases with 9 cases reported during the peak (Fig 1A). However, the shape of the two incidence curves was quite similar (Fig 1A). In addition, cases presenting COVID-19-related clinical phenotypes had the highest cumulative incidence, whereas laboratory-confirmed COVID-19 cases had the lowest cumulative incidence (Fig 1B). The same trends were observed for dental assistants.

**Table 2:** COVID-19-related clinical phenotypes and putative exposure history in dentists.

	All symptomatic dentists	No test performed	Negative test	Positive test	p-value (1)	No COVID-19-related clinical phenotypes	COVID-19-related clinical phenotypes	p-value (2)
	(n = 1097)	(n = 921)	(n = 99)	(n = 77)		(n = 471)	(n = 626)	
**Symptoms**								
Fever (>38°)	350 (31.9)	**269 (29.2)**	**32 (32.3)**	**49 (63.6)**	**<0.001**	**68 (14.4)**	**282 (45.0)**	**<0.001**
Chills	372 (33.9)	321 (34.9)	31 (31.3)	20 (26.0)	0.243	**86 (18.3)**	**286 (45.7)**	**<0.001**
Headache	27 (2.5)	23 (2.5)	3 (3.0)	1 (1.3)	0.751	**19 (4.0)**	**8 (1.3)**	**0.007**
Conjunctivitis	128 (11.7)	104 (11.3)	13 (13.1)	11 (14.3)	0.656	**43 (9.1)**	**85 (13.6)**	**0.029**
Tiredness	796 (72.6)	**650 (70.6)**	**73 (73.7)**	**73 (94.8)**	**<0.001**	**307 (65.2)**	**489 (78.1)**	**<0.001**
Rhinitis	441 (40.2)	363 (39.4)	46 (46.5)	32 (41.6)	0.384	189 (40.1)	252 (40.3)	1
Myalgia	567 (51.7)	**452 (49.1)**	**55 (55.6)**	**60 (77.9)**	**<0.001**	**201 (42.7)**	**366 (58.5)**	**<0.001**
Sore throat	589 (53.7)	496 (53.9)	57 (57.6)	36 (46.8)	0.35	**236 (50.1)**	**353 (56.4)**	**0.045**
Cough	700 (63.8)	582 (63.2)	65 (65.7)	53 (68.8)	0.566	**205 (43.5)**	**495 (79.1)**	**<0.001**
Anosmia	205 (18.7)	**143 (15.5)**	**14 (14.1)**	**48 (62.3)**	**<0.001**	**0 (0.0)**	**205 (32.7)**	**<0.001**
Agueusia	180 (16.4)	**120 (13.0)**	**14 (14.1)**	**46 (59.7)**	**<0.001**	**0 (0.0)**	**180 (28.8)**	**<0.001**
Dyspnea	261 (23.8)	207 (22.5)	29 (29.3)	25 (32.5)	0.057	**85 (18.0)**	**176 (28.1)**	**<0.001**
ARDS	26 (2.4)	**18 (2.0)**	**2 (2.0)**	**6 (7.8)**	**0.005**	8 (1.7)	18 (2.9)	0.286
**Contact history**								
Private sphere								
Spouse	36 (3.3)	28 (3.0)	3 (3.0)	5 (6.5)	0.26	15 (3.2)	21 (3.4)	1
Child	45 (4.1)	41 (4.5)	2 (2.0)	2 (2.6)	0.403	13 (2.8)	32 (5.1)	0.073
Maid	1 (0.1)	1 (0.1)	0 (0.0)	0 (0.0)	0.909	1 (0.2)	0 (0.0)	0.886
Medical appointment	2 (0.2)	2 (0.2)	0 (0.0)	0 (0.0)	0.826	0 (0.0)	2 (0.3)	0.608
During public transportation	25 (2.3)	21 (2.3)	3 (3.0)	1 (1.3)	0.747	11 (2.3)	14 (2.2)	1
During travel	53 (4.8)	48 (5.2)	4 (4.0)	1 (1.3)	0.284	24 (5.1)	29 (4.6)	0.832
Unknown	49 (4.5)	40 (4.3)	2 (2.0)	7 (9.1)	0.071	19 (4.0)	30 (4.8)	0.65
Professional exposure								
Coworker	49 (4.5)	39 (4.2)	5 (5.1)	5 (6.5)	0.626	20 (4.2)	29 (4.6)	0.874
Assistant	38 (3.5)	34 (3.7)	2 (2.0)	2 (2.6)	0.627	17 (3.6)	21 (3.4)	0.951
Secretary	6 (0.5)	4 (0.4)	1 (1.0)	1 (1.3)	0.495	1 (0.2)	5 (0.8)	0.373
Dental procedures	545 (49.7)	**487 (52.9)**	**36 (36.4)**	**22 (28.6)**	**<0.001**	218 (46.3)	327 (52.2)	0.059
Unknown	245 (22.3)	**220 (23.9)**	**17 (17.2)**	**8 (10.4)**	**0.01**	92 (19.5)	153 (24.4)	0.063
PPE								
No specific measures	812 (74.0)	682 (74.0)	78 (78.8)	52 (67.5)	0.24	342 (72.6)	470 (75.1)	0.394
FFP2 mask	96 (8.8)	76 (8.3)	11 (11.1)	9 (11.7)	0.405	44 (9.3)	52 (8.3)	0.622
Safety goggles	680 (62.0)	569 (61.8)	67 (67.7)	44 (57.1)	0.342	284 (60.3)	396 (63.3)	0.349
Hairnets	133 (12.1)	110 (11.9)	15 (15.2)	8 (10.4)	0.578	61 (13.0)	72 (11.5)	0.526
Shoe covers	22 (2.0)	18 (2.0)	2 (2.0)	2 (2.6)	0.928	11 (2.3)	11 (1.8)	0.646
Disposable gown	62 (5.7)	50 (5.4)	7 (7.1)	5 (6.5)	0.755	22 (4.7)	40 (6.4)	0.276

Data are n (%). P-values comparing (1) dentists’ COVID-19 test status (no test, negative or positive) and (2) dentists with a COVID-19-related clinical phenotype vs not, are from Fisher’s exact test. ARDS: acute respiratory distress syndrome; PPE: personal protective equipment. Regarding professional exposure, respondents were asked to define types of dental care and PPE used in the 15 days preceding the onset of symptoms.

### Assessment of risk indicators associated with COVID-19 and COVID-19-related clinical phenotypes in dentists

Because of fewer responses and tested cases for dental assistants, the study focused solely on dentists for assessing risk indicators associated with COVID-19 or COVID-19-related clinical phenotypes.

#### COVID-19

In the univariable analysis, having chronic kidney disease (CKD), being obese, working in a group practice, and maintaining pre-lockdown clinical practice were associated with increased odds of laboratory-confirmed COVID-19, whereas working in a private individual practice and changing clinical practice following lockdown enforcement were associated with decreased odds (Table 3). In the multivariable analysis, history of seasonal allergies, having CKD, and being obese were associated with increased odds of laboratory-confirmed COVID-19, whereas practice limited to endodontics and changing work rhythm or clinical practice were associated with decreased odds (Table 3).

**Table 3:** Risk indicators associated with COVID-19 and COVID-19-related clinical phenotypes in dentists.

	Laboratory-confirmed COVID-19 vs tested negative or not tested		COVID-19-related clinical phenotypes vs not	
	Univariable OR (95% CI, p-value)	Multivariable OR (95% CI, p-value)	Univariable OR (95% CI, p-value)	Multivariable OR (95% CI, p-value)
**Demographic data**				
Age (>44 years)	1.04 (0.66–1.66, p = 0.855)	-	**0.77 (0.64–0.91, p = 0.003)**	0.86 (0.70–1.04, p = 0.120)
Male gender	1.17 (0.75–1.83, p = 0.479)	-	**0.74 (0.62–0.89, p = 0.001)**	**0.74 (0.61–0.89, p = 0.002)**
≥ 1 child	0.72 (0.45–1.14, p = 0.166)	0.66 (0.40–1.05, p = 0.081)	0.96 (0.81–1.14, p = 0.660)	-
**Medical conditions**				
Current pregnancy	1.35 (0.22–4.41, p = 0.676)	-	0.91 (0.46–1.67, p = 0.786)	-
Current smoking	0.99 (0.41–2.03, p = 0.986)	-	1.05 (0.78–1.40, p = 0.740)	-
Comorbidities				
Allergies	3.64 (0.58–12.37, p = 0.081)	**4.61 (0.72–16.52, p = 0.044)**	1.66 (0.66–3.67, p = 0.241)	-
Diabetes	0.75 (0.04–3.45, p = 0.774)	-	0.73 (0.32–1.44, p = 0.400)	-
Hypertension	1.65 (0.72–3.26, p = 0.187)	-	0.89 (0.62–1.26, p = 0.536)	-
Cardiopathies	0.87 (0.14–2.82, p = 0.853)	-	0.87 (0.49–1.44, p = 0.603)	-
COPD	0.66 (0.11–2.14, p = 0.571)	-	**1.61 (1.08–2.35, p = 0.016)**	**1.59 (1.04–2.36, p = 0.027)**
CKD	**6.62 (1.04–23.80, p = 0.013)**	**6.52 (0.95–26.44, p = 0.021)**	1.62 (0.46–4.54, p = 0.395)	-
Malignancies	0.56 (0.03–2.55, p = 0.564)	-	0.60 (0.28–1.14, p = 0.150)	-
Obesity	**2.94 (1.01–6.76, p = 0.023)**	**3.13 (1.06–7.46, p = 0.019)**	1.12 (0.63–1.88, p = 0.678)	-
ID	1.13 (0.06–5.26, p = 0.906)	-	1.54 (0.72–2.99, p = 0.229)	-
Other	1.14 (0.28–3.10, p = 0.826)	-	1.00 (0.61–1.57, p = 0.999)	-
**Clinical practice**				
Specialty				
General practice	0.74 (0.44–1.33, p = 0.289)	-	**0.74 (0.59–0.92, p = 0.006)**	0.82 (0.65–1.04, p = 0.099)
Endodontics	**0.24 (0.04–0.78, p = 0.049)**	**0.21 (0.04–0.69, p = 0.033)**	0.88 (0.65–1.18, p = 0.411)	-
Oral surgery	1.20 (0.64–2.09, p = 0.537)	-	1.18 (0.93–1.47, p = 0.163)	-
Orthodontics	0.48 (0.15–1.16, p = 0.154)	0.39 (0.12–0.95, p = 0.068)	0.98 (0.73–1.29, p = 0.871)	-
Pediatric dentistry	0.52 (0.13–1.39, p = 0.263)	-	**1.43 (1.05–1.92, p = 0.019)**	-
Restaurative dentistry	0.26 (0.04–0.84, p = 0.063)	-	0.92 (0.67–1.24, p = 0.607)	-
Periodontology	0.88 (0.44–1.61, p = 0.707)	-	**1.37 (1.09–1.70, p = 0.005)**	**1.35 (1.06–1.70, p = 0.014)**
Prosthodontics	0.84 (0.41–1.57, p = 0.618)	-	1.02 (0.80–1.29, p = 0.857)	-
Implantology	2.00 (0.69–4.55, p = 0.142)	-	1.38 (0.88–2.09, p = 0.139)	-
Gnathology	2.01 (0.48–5.52, p = 0.246)	-	0.88 (0.44–1.59, p = 0.684)	-
Private practice	**0.49 (0.27–1.00, p = 0.033)**	-	0.76 (0.57–1.04, p = 0.074)	-
Working in group practice	**1.74 (0.98–2.93, p = 0.046)**	-	1.16 (0.91–1.47, p = 0.214)	-
Number of staff				
Medical (>2)	1.40 (0.89–2.19, p = 0.143)	-	**1.20 (1.01–1.42, p = 0.034)**	-
Non-medical (>2)	1.53 (0.98–2.43, p = 0.065)	1.58 (1.00–2.54, p = 0.054)	**1.29 (1.08–1.53, p = 0.004)**	**1.23 (1.02–1.49, p = 0.029)**
Taking public transportation	1.77 (0.95–3.09, p = 0.055)	-	**1.62 (1.27–2.06, p<0.001)**	**1.49 (1.15–1.93, p = 0.002)**
Before lockdown	**2.03 (1.09–3.55, p = 0.018)**	**1.86 (0.97–3.33, p = 0.046)**	**1.58 (1.22–2.03, p<0.001)**	-
After lockdown	0.93 (0.23–2.52, p = 0.900)	-	**1.67 (1.14–2.40, p = 0.007)**	-
**Changes after lockdown**				
Private sphere				
Household size increase	0.65 (0.30–1.23, p = 0.219)	-	**0.68 (0.53–0.88, p = 0.003)**	0.77 (0.58–1.00, p = 0.057)
Household size decrease	1.04 (0.36–2.35, p = 0.935)	-	1.38 (0.99–1.89, p = 0.053)	1.34 (0.94–1.87, p = 0.093)
Relocation	0.60 (0.10–1.92, p = 0.477)	-	**1.53 (1.03–2.21, p = 0.027)**	-
Other	3.40 (0.55–11.52, p = 0.097)	-	**2.49 (1.13–5.11, p = 0.017)**	2.19 (0.92–4.85, p = 0.061)
Work environment				
No change	**2.26 (1.12–4.16, p = 0.014)**	-	**1.57 (1.16–2.11, p = 0.003)**	-
Work rhythm	0.66 (0.39–1.08, p = 0.108)	**0.58 (0.34–0.96, p = 0.039)**	**0.56 (0.46–0.68, p<0.001)**	**0.51 (0.41–0.63, p<0.001)**
Clinical practice	**0.33 (0.21–0.52, p<0.001)**	**0.32 (0.20–0.51, p<0.001)**	**0.74 (0.61–0.88, p = 0.001)**	**0.73 (0.61–0.89, p = 0.002)**
Reduce number of medical staff	1.00 (0.54–1.71, p = 0.992)	-	0.87 (0.69–1.08, p = 0.219)	-
Reduce number of paramedical staff	0.90 (0.55–1.43, p = 0.651)	-	**0.74 (0.61–0.89, p = 0.001)**	-
Reduce number of administrative staff	1.24 (0.73–2.04, p = 0.409)	-	0.84 (0.68–1.04, p = 0.120)	-
Work stopping	**2.99 (1.23–6.19, p = 0.007)**	-	**1.86 (1.23–2.75, p = 0.002)**	-

OR = odds ratio; 95% CI = 95% confident interval. COPD: chronic obstructive pulmonary disease; CKD: chronic kidney disease; ID: immunodeficiencies.

Among symptomatic dentists, in the univariable analysis, having fever, tiredness, myalgia, anosmia, agueusia and acute respiratory distress syndrome were associated with increased odds of laboratory-confirmed COVID-19, whereas performing dental procedures was associated with decreased odds (Table 4). In the multivariable analysis, only performing dental procedures was associated with decreased odds of laboratory-confirmed COVID-19 (Table 4).

**Table 4:** Risk indicators associated with COVID-19 and COVID-19-related clinical phenotypes in symptomatic dentists.

	Laboratory-confirmed COVID-19 vs tested negative or not tested		COVID-19-related clinical phenotypes vs not		
	Univariable OR (95% CI, p-value)	Multivariable OR (95% CI, p-value)	Univariable OR (95% CI, p-value)	Multivariable OR (95% CI, p-value)	
**Symptom**					
Fever (>38°C)	**4.18 (2.60–6.85, p<0.001)**	-	**4.86 (3.61–6.61, p<0.001)**	-	
Chills	0.67 (0.38–1.11, p = 0.129)	-	**3.77 (2.85–5.01, p<0.001)**	-	
Headache	0.50 (0.03–2.42, p = 0.503)	-	**0.31 (0.13–0.69, p = 0.006)**	-	
Conjunctivitis	1.29 (0.63–2.41, p = 0.459)	-	**1.56 (1.07–2.32, p = 0.024)**	-	
Tiredness	**7.50 (3.08–24.77, p<0.001)**	-	**1.91 (1.46–2.50, p<0.001)**	-	
Rhinitis	1.06 (0.66–1.69, p = 0.801)	-	1.01 (0.79–1.28, p = 0.966)	-	
Myalgia	**3.57 (2.10–6.39, p<0.001)**	-	**1.89 (1.49–2.41, p<0.001)**	-	
Sore throat	0.74 (0.46–1.18, p = 0.207)	-	**1.29 (1.01–1.64, p = 0.039)**	-	
Cough	1.27 (0.78–2.13, p = 0.343)	-	**4.90 (3.77–6.41, p<0.001)**	-	
Anosmia	**9.10 (5.60–15.02, p<0.001)**	-		-	
Agueusia	**9.81 (6.04–16.15, p<0.001)**	-		-	
Dyspnea	1.60 (0.96–2.60, p = 0.066)	-	**1.78 (1.33–2.39, p<0.001)**	-	
ARDS	**4.23 (1.51–10.28, p = 0.003)**	-	1.71 (0.76–4.21, p = 0.210)	-	
**Contact history**					
Private sphere					
Spouse	2.22 (0.74–5.41, p = 0.110)	1.91 (0.59–5.26, p = 0.239)	1.06 (0.54–2.11, p = 0.876)	1.23 (0.61–2.55, p = 0.569)	
Child	0.61 (0.10–2.02, p = 0.494)	-	1.90 (1.01–3.79, p = 0.056)	-	
During public transportation	0.55 (0.03–2.64, p = 0.556)	-	0.96 (0.43–2.17, p = 0.913)	-	
During travel	0.24 (0.01–1.14, p = 0.166)	0.19 (0.01–0.92, p = 0.104)	0.90 (0.52–1.59, p = 0.723)	1.05 (0.57–1.94, p = 0.872)	
Other	**2.33 (0.93–5.07, p = 0.048)**	1.90 (0.69–4.77, p = 0.188)	1.20 (0.67–2.19, p = 0.548)	1.41 (0.75–2.71, p = 0.287)	
Work environment					
Coworker	1.54 (0.52–3.67, p = 0.375)	-	1.10 (0.62–1.99, p = 0.759)	-	
Assistant	0.73 (0.12–2.45, p = 0.668)	-	0.93 (0.48–1.80, p = 0.819)	-	
Secretary	2.67 (0.14–16.84, p = 0.373)	-	3.78 (0.61–72.63, p = 0.225)	-	
Unknown	**0.38 (0.17–0.76, p = 0.012)**	0.68 (0.27–1.59, p = 0.382)	1.33 (1.00–1.79, p = 0.054)	1.21 (0.86–1.70, p = 0.286)	
**Professional exposure**					
Dental procedures	**0.38 (0.22–0.62, p<0.001)**	**0.39 (0.20–0.74, p = 0.005)**	1.27 (1.00–1.61, p = 0.051)	1.20 (0.86–1.66, p = 0.278)	
PPE					
No specific measures	0.71 (0.44–1.19, p = 0.180)	1.39 (0.73–2.79, p = 0.330)	1.14 (0.87–1.49, p = 0.356)	1.05 (0.71–1.54, p = 0.805)	
FFP2 mask	1.42 (0.64–2.80, p = 0.346)	-	0.88 (0.58–1.34, p = 0.548)	-	
Safety goggles	0.81 (0.51–1.29, p = 0.365)	-	1.13 (0.89–1.45, p = 0.317)	-	
Hairnets	0.83 (0.36–1.67, p = 0.629)	-	0.87 (0.61–1.26, p = 0.467)	-	
Shoe covers	1.33 (0.21–4.69, p = 0.702)	-	0.75 (0.32–1.76, p = 0.500)	-	
Disposable gown	1.17 (0.40–2.75, p = 0.740)	-	1.39 (0.82–2.41, p = 0.224)	-	

OR = odds ratio; 95% CI = 95% confident interval. ARDS: acute respiratory distress syndrome; PPE: personal protective equipment. Regarding professional exposure, respondents were asked to define types of dental care and PPE used in the 15 days preceding the onset of symptoms.

#### COVID-19-related clinical phenotypes

Data regarding COVID-19-related phenotypes are given in Table 3. In the univariable analysis, odds of presenting a COVID-19-related clinical phenotype were higher in younger dentists, females, dentists with chronic obstructive pulmonary disease (COPD), users of public transportation, dentists with a practice limited to pediatric dentistry or periodontology, and dentists who maintained their pre-lockdown clinical practice, whereas odds were lower for dentists working within a general practice, those having a practice limited to endodontics, who changed their work rhythm or clinical practice and who reduced the number of non-medical staff in their practice. In the multivariable analysis, female gender, COPD, use of public transportation, and having a practice limited to periodontology were associated with increased odds of having a COVID-19-related clinical phenotype, whereas changing one’s work rhythm or clinical practice were both associated with decreased odds.

### Perceived stress

Overall, alongside concerns regarding contaminating their families (median NRS score = 6 [IQR, 4 to 8]), dentists were more anxious about current or future financial and organizational difficulties in their professional practice (7 [5 to 8]) than to be contaminated (4 [2–6]). Regarding type of professional practice, private practitioners were more anxious (7 [5–9] vs 5 [2–7], p<0.001) whereas those who worked in group practices were less anxious (6 [3–8] vs 8 [5–9]), p<0.001). Compared to dentists, dental assistants had higher median NRS scores on stress pertaining to family (7 [5–9]) and personal safety (5 [3–7]) (see S1 Table).

## Discussion

To the best of our knowledge, this large survey is the first study to assess the impact of the COVID-19 pandemic and associated clinical phenotypes amongst oral health-care workers. Our sample of dentists was representative in terms of mean age (45 years for our sample vs. 47 for the general oral population) and clinical practice (92 for our sample worked in a private practice vs. 87 for the general oral population), suggesting that for these variables the sampling bias was minimal. However, 43% of the sample were men compared to 54% within the general dental population, but a feminization of the profession is underway, according to the ONDPS (http://www.ordre-chirurgiens-dentistes.fr/cartographie/).

Results from this study suggest that at time of data collection (April 29, 2020), the prevalence of laboratory-confirmed COVID-19 among dentists was 1.9%, similar to that of the French population at the same date (2%, www.santepubliquefrance.fr). Although the prevalence among dental assistants was lower mainly due to a low testing rate, they seem to be as often infected as dentists, with a similar proportion of laboratory-confirmed COVID-19 cases among tested participants in both groups (about 39%). After analysis with a highly specific predictive model, it is probable that such prevalences are underestimated (double or triple respectively). This is in adherence with the high prevalence of COVID-19-related clinical phenotypes observed both in dentists (15.0%) and dental assistants (11.8%). The prevalence of symptomatic dentists was similar to that of the French population at the same date (14%, www.santepubliquefrance.fr). Possible explanations for such underestimation are twofold: (1) very few people have been tested in this sample (<5%), (2) depending on timing of exposure and symptom onset, the false-negative rate of viral tests varies from 20% to 100% [22], and (3) testing varied greatly depending on patient profile. Indeed, only symptomatic subjects or those exhibiting specific comorbidities such as allergies or obesity were associated with increased odds of being tested in this study. This is consistent with the French government policy to test only symptomatic people or those with risk factors of developing severe COVID-19 (www.santepubliquefrance.fr/maladies-et-traumatismes/maladies-et-infections-respiratoires/infection-a-coronavirus/documents/affiche/alerte-coronavirus-les-tests-de-depistage-ne-sont-pas-automatiques-affiche-a4-francais), except for seasonal allergies that surprisingly did not seem to be considered [23, 24]. Furthermore, odds of being tested were also higher in people living with children, possibly explained by an increased fear of disseminating SARS-CoV-2 in their homes, as reported in previous international studies [25], that could have led them to get tested even without fulfilling the French government testing policy requirements. In addition, working in group practices seemed associated with increased odds of being tested, possibly due to the easier implementation of SARS-CoV-2 testing in larger structures, compared to private practices with fewer staff. Nevertheless, this variable was no longer significant in the multivariable analysis, possibly explained by a phenomenon of multicollinearity with general practitioners, who would less often work in group practices (OR 0.22 IC95% [0.18 to 0.27], p<0.001) and seemed to be associated with decreased odds of being tested. Currently, the French government testing policy has changed, and health-care workers have easier access to testing, including serological tests. Therefore, a three-month follow-up questionnaire for French oral health-care workers is ongoing. It will also allow us to assess if clinical practices have changed since the end of the first-wave pandemic, in particular types of PPE used.

The peak of outbreak was observed between March 16 and March 18, 2020 for all groups of respondents, with three quarters of symptoms occurring between March 20 and 22. Therefore most cases of COVID-19 occurred before French national lockdown, considering a mean incubation period of 3 to 5 days for SARS-CoV-2 (as for other respiratory viruses such as SARS or MERS) [26–29]. Although lockdown and home confinement have had an indisputable mitigating effect, it is probable that, had they been enforced two weeks earlier, a high number of contaminations could have been prevented. In addition, most symptomatic respondents suspected a work-related contamination. Indeed, adequate specific PPE (FFP2 mask, safety goggles…) were only enforced after nationwide lockdown (i.e. too late), leading to dentists treating patients without adequate PPE in the weeks prior to lockdown. Moreover, dental assistants were similarly exposed during dental care but used specific PPE even less often than dentists.

Comorbidities such as chronic kidney disease and obesity were the only risk indicators associated with increased odds of laboratory-confirmed COVID-19, in adherence with risk factors identified in previous studies [30, 31]. Conversely, having a practice limited to endodontics was associated with decreased odds of laboratory-confirmed COVID-19, possibly explained by the regular use of rubber dam isolation for endodontics procedures, which could drastically reduce the salivary content of instrument-generated aerosols (i.e. at least a 70% decrease) [32, 33]. At the time of survey, respondents who performed dental procedures were paradoxically better protected against COVID-19 than those who did not. A possible explanation to such paradox could be found in the fact that surgical masks were not recommended at that time for the general population whereas dentists wore surgical masks (and other personal protective equipment) as part of their routine practice, especially during dental procedures. Thus, performing dental procedures (and subsequent wear of personal protective equipment) could have been protective regarding the risk of COVID-19 development, as compared to those who did not perform such procedures and who were thus most likely to wear surgical masks infrequently. When comparing symptomatic cases, it was not possible to identify any conventional PPE (surgical mask and gloves) or specific PPE (FFP2 mask, safety goggles, hairnets, shoe covers or disposable gown) as protective indicators against COVID-19, possibly explained by similar transmission routes with other respiratory viruses that could account for the COVID-19-related clinical phenotypes [34]. In reality, this variable was strongly associated with practice limited to oral surgery (3.23 [1.80 to 5.69], p< 0.001) and practice limited to periodontology (2.69 [1.49 to 4.74], p< 0.001), both being confounding variables. Indeed, working in a practice limited to periodontology was associated with higher odds of presenting a COVID-19-related clinical phenotype. Consequently, working in dental specialties highly exposed to airborne droplets such as periodontology, would appear to be an at-risk practice, with subsequent adjustments in necessary protective equipment. Further studies are needed to confirm this assumption.

Exploration of risk indicators associated with COVID-19-related clinical phenotypes (that could be observed in other similar respiratory infections) found that using public transportation was associated with increased odds of presenting a COVID-19-related clinical phenotype, in adherence with previous results showing an increased risk of respiratory viruses transmission due to proximity in a closed environment [35]. Moreover, having a practice limited to periodontology was associated with higher odds of presenting a COVID-19-related clinical phenotype. Indeed, periodontists routinely use ultrasonic devices, causing saliva projections and aerosols, major transmission routes of respiratory viruses [36, 37]. Consequently, future guidelines should focus on the usage of ultrasonic devices and the adaptation of PPE to the type of clinical practice. For instance, specific PPE usage should be encouraged on a regular basis in periodontology. This could probably apply to other specialties such as pediatric dentistry, although this variable was significant only in the univariable analysis. Female gender could be another risk indicator of presenting a COVID-19-related clinical phenotype. Although older men are more frequently infected with SARS-CoV-2, people of all ages and all genders are susceptible to respiratory viral infections, and younger people have indeed been infected with SARS-CoV, H1N1 or H5N1 [38–40].

Finally, changing one’s work rhythm or one’s clinical practice were both associated with decreased odds of both laboratory-confirmed COVID-19 and presenting a COVID-19-related clinical phenotype. Thus, the reduction of dental activity during lockdown, for example by participating in telephone regulation of emergency cases or limiting one’s practice to emergency cases only, as recommended by French authorities, seems to have protected dentists from COVID-19. This could also explain why they were not more contaminated than the general population.

Apart from the aforementioned symptomatology and morbidity, the COVID-19 pandemic also had a strong negative psychological impact on oral health-care workers. Indeed, dentists and dental assistants reported specific anxiety regarding their professional activity (and prospects), in particular for private practices, possibly explained by the sharp decline in attendance, which reached a 94% decrease in April 2020 [41, 42]. Moreover, significant concerns arose regarding transmitting the infection to their families, as previously mentioned for other health-care workers [43].

Several limitations must be considered in this study and its methodology. First, an open form of recruitment was used (Google Forms^®^), that does not control who answers. However, the survey was distributed solely through professional channels. Other limitations of Google Forms^®^ such as the possibility to answer several times were considered by the authors but it was judged that there was very little incentive for users to answer more than once, and this was further controlled by inspecting the age and initials fields for doubles. Although Google Forms^®^ it is not as feature-rich as fully customizable commercial software, it provided interesting functionalities such as skip logic, conditional formatting and different question types that were required for this study. Second, a non-random sampling method was used, that does not guarantee a representative sample of the study population [44]. For instance, respondents who participated could have been those who felt the most concerned, i.e. those infected with SARS-CoV-2. Third, some participants may have overdeclared their symptomatology, due to social media-fueled panic, as previously reported [45]. Conversely, since the survey assessed self-reported data, a recall bias is a possibility as participants may not remember exposure and contact history accurately or may omit details. However, as the events were recalled after a short time interval within a population concerned about this pandemic, we believe this bias to be minimal at best. Fourth, oral health-care workers who had died before the survey started or those who had been hospitalized were obviously not included, leading to an underestimation of the measure of association [46]. Fifth, risk of differential bias may be high when comparing people with a laboratory-confirmation of COVID-19 and those tested negative or not tested. Indeed, some non-tested people could be positive for COVID-19 and other tested negative people could actually be infected. However, a predictive model was developed as a sensitivity analysis to assess impact of this potential bias on results. Except for BCG vaccination, the predictive model did not yield specific variables.

In conclusion, results from the present study suggest that although oral health-care professionals were surprisingly not at higher risk of COVID-19 than the general population, specific risk indicators could exist, notably among high aerosol-generating dental subspecialties such as periodontology. Considering the similarities between COVID-19-related clinical phenotypes and those of other viral respiratory infections, lessons can be learned from the COVID-19 pandemic regarding the usefulness of equipping and protecting oral health-care workers, notably during seasonal viral outbreaks, to limit infection spread. In fine, protecting oral health-care workers could thus be an interesting public health strategy to prevent the resurgence of COVID-19 and/or the emergence of new pandemics.

## Supporting information

S1 Table Socio-demographic data, health status, clinical practice, changes after enforcement of home confinement, perceived stress, and COVID-19 status in all respondents. (DOCX)

S2 Table Socio-demographic data, health status, clinical practice and COVID-19 status in dentists. (DOCX)

S3 Table Risk indicators of being tested for COVID-19 in dentists. (DOCX)

S4 Table Clinical phenotypes associated with COVID-19 among dentists. (DOCX)

S5 Table Putative exposure history in all symptomatic respondents. (DOCX)

S1 Fig (PDF)

S1 Data (XLSX)
