# Supplementary material for: Prevalence and risk indicators of first-wave COVID-19 among oral health-care workers: A French epidemiological survey
Source: PLoS One. 2021 Feb 11;16(2):e0246586. doi: 10.1371/journal.pone.0246586 (PMC7877573; doi:10.1371/journal.pone.0246586)
Supplement: S1 Table — (DOCX) [file pone.0246586.s001.docx]

|  | **Dental assistants (N=1868)** | **Dentists (N=4172)** | **p-value** |
| --- | --- | --- | --- |
| **Demographic data** |  |  |  |
| Age, years | **38.00 [31.00, 46.00]** | **44.00 [34.00, 55.00]** | **<0.001**^#^ |
| Male gender | **33 (1.8)** | **1791 (42.9)** | **<0.001** |
| Household size | 3.00 [2.00, 4.00] | 3.00 [2.00, 4.00] | 0.139^#^ |
| ≥ 1 child | **712 (38.1)** | **1853 (44.4)** | **<0.001** |
| **Medical conditions** |  |  |  |
| Current pregnancy | 25 (1.3) | 79 (1.9) | 0.154 |
| Current smoking | **383 (20.5)** | **372 (8.9)** | **<0.001** |
| Comorbidity |  |  |  |
| Allergies | 18 (1.0) | 31 (0.7) | 0.467 |
| Diabetes | 25 (1.3) | 70 (1.7) | 0.385 |
| Hypertension | **91 (4.9)** | **270 (6.5)** | **0.018** |
| Cardiopathies | **36 (1.9)** | **120 (2.9)** | **0.039** |
| COPD | **106 (5.7)** | **156 (3.7)** | **0.001** |
| CKD | 9 (0.5) | 18 (0.4) | 0.95 |
| Malignancies | **60 (3.2)** | **93 (2.2)** | **0.031** |
| Obesity | **86 (4.6)** | **97 (2.3)** | **<0.001** |
| ID | 29 (1.6) | 47 (1.1) | 0.212 |
| Other | 75 (4.0) | 140 (3.4) | 0.229 |
| BCG vaccination |  |  | **<0.001** |
| Coverage | 1522 (81.5) | 3151 (75.5) |  |
| No coverage | 59 (3.2) | 202 (4.8) |  |
| Unknown | 287 (15.4) | 819 (19.6) |  |
| **Clinical practice** |  |  |  |
| Specialty |  |  |  |
| General practice | **1500 (80.3)** | **3508 (84.1)** | **<0.001** |
| Endodontics | **563 (30.1)** | **397 (9.5)** | **<0.001** |
| Oral surgery | **596 (31.9)** | **636 (15.2)** | **<0.001** |
| Orthodontics | **394 (21.1)** | **414 (9.9)** | **<0.001** |
| Pediatric dentistry | **167 (8.9)** | **294 (7.0)** | **0.012** |
| Restorative dentistry | **358 (19.2)** | **369 (8.8)** | **<0.001** |
| Periodontology | **588 (31.5)** | **644 (15.4)** | **<0.001** |
| Prosthodontics | **724 (38.8)** | **610 (14.6)** | **<0.001** |
| Implantology | **173 (9.3)** | **139 (3.3)** | **<0.001** |
| Gnathology | 49 (2.6) | 82 (2.0) | 0.127 |
| Disability | 2 (0.1) | 6 (0.1) | 1 |
| Other | **0 (0.0)** | **27 (0.6)** | **0.001** |
| Private practice | **1643 (88.0)** | **3858 (92.5)** | **<0.001** |
| Working in group practice | **201 (10.8)** | **574 (13.8)** | **0.001** |
| Number of staff |  |  |  |
| Medical | **2.00 [2.00, 3.00]** | **2.00 [2.00, 4.00]** | **0.025**^#^ |
| Non-medical | **3.00 [2.00, 5.00]** | **2.00 [1.00, 4.00]** | **<0.001**^#^ |
| **Changes after lockdown** |  |  |  |
| Taking public transportation | **317 (17.0)** | **457 (11.0)** | **<0.001** |
| Before lockdown | **295 (15.8)** | **406 (9.7)** | **<0.001** |
| After lockdown | **122 (6.5)** | **170 (4.1)** | **<0.001** |
| Family environment |  |  |  |
| No change | **1544 (82.7)** | **3063 (73.4)** | **<0.001** |
| Household size increase | **178 (9.5)** | **689 (16.5)** | **<0.001** |
| Household size decrease | 101 (5.4) | 255 (6.1) | 0.309 |
| Relocation | **47 (2.5)** | **172 (4.1)** | **0.003** |
| Other | **0 (0.0)** | **33 (0.8)** | **<0.001** |
| Work environment |  |  |  |
| No change | **175 (9.4)** | **284 (6.8)** | **0.001** |
| Change in workplace location | 0 (0.0) | 7 (0.2) | 0.173 |
| Change in work rhythm | **184 (9.9)** | **1412 (33.8)** | **<0.001** |
| Change in clinical practice | **183 (9.8)** | **2966 (71.1)** | **<0.001** |
| Reduced number of medical staff | **59 (3.2)** | **794 (19.0)** | **<0.001** |
| Reduced number of non-medical staff | **60 (3.2)** | **2318 (55.5)** | **<0.001** |
| Work stopping | **1452 (77.7)** | **136 (3.3)** | **<0.001** |
| **Perceived stress** |  |  |  |
| Personal safety | **5 [3, 7]** | **4 [2, 6]** | **<0.001**^#^ |
| Family safety | **7 [5, 9]** | **6 [4, 8]** | **<0.001**^#^ |
| Clinical practice | 7 [5, 9] | 7 [5, 8] | 0.491^#^ |
| **Test for COVID-19** |  |  | **<0.001** |
| Negative | 22 (1.2) | 120 (2.9) |  |
| None | 1832 (98.1) | 3973 (95.2) |  |
| Positive | 14 (0.7) | 79 (1.9) |  |

Data are median [IQR], n (%). p values comparing dental assistants and dental practitioners are from (#) Mann-Whitney U test or Fisher’s exact test when not specified. COPD: chronic obstructive pulmonary disease; CKD: chronic kidney disease; ID: immunodeficiencies.

**Table S1. Socio-demographic data, health status, clinical practice, changes after enforcement of home confinement, perceived stress, and COVID-19 status in all respondents**
