# Supplementary material for: Prevalence and risk indicators of first-wave COVID-19 among oral health-care workers: A French epidemiological survey
Source: PLoS One. 2021 Feb 11;16(2):e0246586. doi: 10.1371/journal.pone.0246586 (PMC7877573; doi:10.1371/journal.pone.0246586)
Supplement: S2 Table — (DOCX) [file pone.0246586.s002.docx]

|  | **All included dentists (N=4172)** | **(1) Not tested for COVID-19 (n=3973)** | **(2) Tested for COVID-19 (n=199)** | **p value (1 vs 2)** |
| --- | --- | --- | --- | --- |
| **Demographic data** |  |  |  |  |
| Age, years | 44.00 [34.00, 55.00] | 44.00 [34.00, 55.00] | 44.00 [35.00, 53.75] | 0.305^#^ |
| Male gender | 1791 (42.9) | 1710 (43.0) | 81 (40.7) | 0.564 |
| Household size | 3.00 [2.00, 4.00] | 3.00 [2.00, 4.00] | 3.00 [2.00, 4.00] | 0.233^#^ |
| ≥ 1 child | 1853 (44.4) | **1780 (44.8)** | **73 (36.7)** | **0.03** |
| **Medical conditions** |  |  |  |  |
| Current pregnancy | 79 (1.9) | 75 (1.9) | 4 (2.0) | 1 |
| Current smoking | 372 (8.9) | 358 (9.0) | 14 (7.0) | 0.408 |
| Comorbidity |  |  |  |  |
| Allergies | 31 (0.7) | 27 (0.7) | 4 (2.0) | 0.087 |
| Diabetes | 70 (1.7) | 69 (1.7) | 1 (0.5) | 0.298 |
| Hypertension | 270 (6.5) | 255 (6.4) | 15 (7.5) | 0.632 |
| Cardiopathies | 120 (2.9) | 114 (2.9) | 6 (3.0) | 1 |
| COPD | 156 (3.7) | 145 (3.6) | 11 (5.5) | 0.242 |
| CKD | 18 (0.4) | 15 (0.4) | 3 (1.5) | 0.069 |
| Malignancies | 93 (2.2) | 91 (2.3) | 2 (1.0) | 0.341 |
| Obesity | 97 (2.3) | 88 (2.2) | 9 (4.5) | 0.062 |
| ID | 47 (1.1) | 45 (1.1) | 2 (1.0) | 1 |
| Other | 140 (3.4) | 133 (3.3) | 7 (3.5) | 1 |
| BCG vaccination |  |  |  | 0.465 |
| Coverage | 3151 (75.5) | 3006 (75.7) | 145 (72.9) |  |
| No coverage | 202 (4.8) | 189 (4.8) | 13 (6.5) |  |
| Unknown | 819 (19.6) | 778 (19.6) | 41 (20.6) |  |
| **Clinical practice** |  |  |  |  |
| Specialty |  |  |  |  |
| General practice | 3508 (84.1) | **3352 (84.4)** | **156 (78.4)** | **0.032** |
| Endodontics | 397 (9.5) | 383 (9.6) | 14 (7.0) | 0.272 |
| Oral surgery | 636 (15.2) | 599 (15.1) | 37 (18.6) | 0.213 |
| Orthodontics | 414 (9.9) | 400 (10.1) | 14 (7.0) | 0.202 |
| Pediatric dentistry | 294 (7.0) | 279 (7.0) | 15 (7.5) | 0.892 |
| Restorative dentistry | 369 (8.8) | 353 (8.9) | 16 (8.0) | 0.778 |
| Periodontology | 644 (15.4) | 605 (15.2) | 39 (19.6) | 0.118 |
| Prosthodontics | 610 (14.6) | 579 (14.6) | 31 (15.6) | 0.773 |
| Implantology | 139 (3.3) | 128 (3.2) | 11 (5.5) | 0.117 |
| Disability | 82 (2.0) | 76 (1.9) | 6 (3.0) | 0.406 |
| Gnathology | 6 (0.1) | 6 (0.2) | 0 (0.0) | 1 |
| Other | 27 (0.6) | 24 (0.6) | 3 (1.5) | 0.272 |
| Private practice | 3858 (92.5) | 3680 (92.6) | 178 (89.4) | 0.128 |
| Working in group practice | 574 (13.8) | **536 (13.5)** | **38 (19.1)** | **0.033** |
| Number of staff |  |  |  |  |
| Medical | 2.00 [2.00, 4.00] | **2.00 [2.00, 4.00]** | **3.00 [2.00, 5.00]** | **0.004**^#^ |
| Non-medical | 2.00 [1.00, 4.00] | **2.00 [1.00, 4.00]** | **3.00 [2.00, 6.00]** | **0.003**^#^ |
| Taking public transportation | 457 (11.0) | **425 (10.7)** | **32 (16.1)** | **0.024** |
| **COVID-19 status** |  |  |  |  |
| ≥ 1 symptom | 1097 (26.3) | **921 (23.2)** | **176 (88.4)** | **<0.001** |
| Test for COVID-19 |  |  |  | **<0.001** |
| Positive | 79 (1.9) | 0 (0.0) | 79 (39.7) |  |
| Negative | 120 (2.9) | 0 (0.0) | 120 (60.3) |  |
| None | 3973 (95.2) | 3973 (100.0) | 0 (0.0) |  |

Data are median [IQR], n (%). p values comparing dentists tested for COVID-19 and not tested (#) Mann-Whitney U test, (*) Kruskal-Wallis or Fisher’s exact test when not specified. COPD: chronic obstructive pulmonary disease; CKD: chronic kidney disease; ID: immunodeficiencies.

**Table S2. Socio-demographic data, health status, clinical practice and COVID-19 status in dentists**
