# Supplementary material for: Prevalence and risk indicators of first-wave COVID-19 among oral health-care workers: A French epidemiological survey
Source: PLoS One. 2021 Feb 11;16(2):e0246586. doi: 10.1371/journal.pone.0246586 (PMC7877573; doi:10.1371/journal.pone.0246586)
Supplement: S3 Table — (DOCX) [file pone.0246586.s003.docx]

|  | **Tested for COVID-19 vs not tested** | |
| --- | --- | --- |
|  | **Univariable OR (95% CI, p-value)** | **Multivariable OR (95% CI, p-value)** |
| **Demographic data** |  |  |
| Age (> 44 years) | 0.98 (0.73-1.32, p=0.918) | - |
| Male gender | 0.91 (0.68-1.21, p=0.516) | - |
| Household size (>3) | 1.14 (0.85-1.52, p=0.370) | - |
| ≥ 1 child | **0.71 (0.53-0.96, p=0.025)** | **0.66 (0.49-0.90, p=0.008)** |
| **Medical conditions** |  |  |
| Current pregnancy | 1.07 (0.32-2.60, p=0.902) | - |
| Current smoking | 0.76 (0.42-1.28, p=0.341) | - |
| Comorbidity | **3.00 (0.88-7.76, p=0.042)** | - |
| Allergies | 0.29 (0.02-1.30, p=0.215) | **3.60 (1.04-9.48, p=0.020)** |
| Diabetes | 1.19 (0.66-1.98, p=0.532) | - |
| Hypertension | 1.05 (0.41-2.23, p=0.904) | - |
| Cardiopathies | 1.54 (0.78-2.77, p=0.176) | - |
| COPD | **4.04 (0.93-12.36, p=0.028)** | 1.55 (0.77-2.80, p=0.182) |
| CKD | 0.43 (0.07-1.38, p=0.244) | 3.17 (0.71-10.03, p=0.077) |
| Malignancies | **2.09 (0.97-4.00, p=0.039)** | - |
| Obesity | 0.89 (0.14-2.89, p=0.868) | **2.29 (1.05-4.45, p=0.023)** |
| ID | 1.05 (0.44-2.12, p=0.897) | - |
| Other | 1.07 (0.32-2.60, p=0.902) | - |
| BCG vaccination |  |  |
| Coverage | 0.92 (0.65-1.32, p=0.626) | - |
| No coverage | 1.31 (0.66-2.42, p=0.417) | - |
| **Clinical practice** |  |  |
| Specialty |  |  |
| General practice | **0.67 (0.48-0.96, p=0.025)** | **0.66 (0.44-1.01, p=0.049)** |
| Endodontics | 0.71 (0.39-1.19, p=0.224) | - |
| Oral surgery | 1.29 (0.88-1.84, p=0.179) | 1.03 (0.66-1.55, p=0.906) |
| Orthodontics | 0.68 (0.37-1.13, p=0.165) | **0.51 (0.27-0.90, p=0.028)** |
| Pediatric dentistry | 1.08 (0.60-1.79, p=0.782) | - |
| Restorative dentistry | 0.90 (0.51-1.46, p=0.682) | - |
| Periodontology | 1.36 (0.93-1.93, p=0.097) | 1.16 (0.75-1.74, p=0.492) |
| Prosthodontics | 1.08 (0.72-1.58, p=0.696) | - |
| Implantology | 1.76 (0.88-3.17, p=0.081) | 1.49 (0.73-2.76, p=0.240) |
| Gnathology | 1.59 (0.61-3.41, p=0.279) | - |
| Other | 2.52 (0.59-7.28, p=0.134) | 1.62 (0.36-5.07, p=0.456) |
| Private practice | 0.67 (0.43-1.11, p=0.099) | 1.13 (0.55-2.32, p=0.742) |
| Working in group practice | **1.51 (1.04-2.16, p=0.026)** | 1.40 (0.60-3.04, p=0.410) |
| Number of staff |  |  |
| Medical (>2) | 1.26 (0.95-1.68, p=0.112) | 1.00 (0.69-1.44, p=0.984) |
| Non-medical (>2) | **1.35 (1.02-1.80, p=0.039)** | 1.20 (0.82-1.74, p=0.343) |
| Taking public transportation | **1.60 (1.06-2.33, p=0.019)** | **1.47 (0.95-2.21, p=0.074)** |

OR=odds ratio; 95% CI= 95% confident interval. COPD: chronic obstructive pulmonary disease; CKD: chronic kidney disease; ID: immunodeficiencies.

**Table S3. Risk indicators of being tested for COVID-19 in dentists**
