# Supplementary material for: Prevalence and risk indicators of first-wave COVID-19 among oral health-care workers: A French epidemiological survey
Source: PLoS One. 2021 Feb 11;16(2):e0246586. doi: 10.1371/journal.pone.0246586 (PMC7877573; doi:10.1371/journal.pone.0246586)
Supplement: S4 Table — (DOCX) [file pone.0246586.s004.docx]

| **Clinical phenotypes associated with COVID-19** | **All included dentists (n=4172)** | **No test performed (n=3973)** | **Tested Negative (n=120)** | **Tested Positive (n=79)** | **p-value** |
| --- | --- | --- | --- | --- | --- |
| Fever and cough | 238 (5.7) | 178 (4.5) | 24 (20.0) | 36 (45.6) | <0.001 |
| Anosmia or agueusia | 245 (5.9) | 174 (4.4) | 18 (15.0) | 53 (67.1) | <0.001 |
| Ageusia and fever or cough | 137 (3.3) | 85 (2.1) | 11 (9.2) | 41 (51.9) | <0.001 |
| Chills and fever or cough | 262 (6.3) | 223 (5.6) | 21 (17.5) | 18 (22.8) | <0.001 |
| Cough and anosmia or agueusia | 154 (3.7) | 104 (2.6) | 12 (10.0) | 38 (48.1) | <0.001 |
| Anosmia and fever or cough | 157 (3.8) | 105 (2.6) | 10 (8.3) | 42 (53.2) | <0.001 |
| Cough and agueusia | 115 (2.8) | 73 (1.8) | 9 (7.5) | 33 (41.8) | <0.001 |
| Cough and chills | 249 (6.0) | 214 (5.4) | 20 (16.7) | 15 (19.0) | <0.001 |
| Fever and anosmia or agueusia | 108 (2.6) | 69 (1.7) | 6 (5.0) | 33 (41.8) | <0.001 |
| Digestive symptoms | 1 (0.0) | 1 (0.0) | 0 (0.0) | 0 (0.0) | 0.975 |
| Agueusia and myalgia or tiredness | 157 (3.8) | 102 (2.6) | 11 (9.2) | 44 (55.7) | <0.001 |
| Fever and agueusia | 83 (2.0) | 49 (1.2) | 5 (4.2) | 29 (36.7) | <0.001 |
| Cough and digestive symptoms | 30 (0.7) | 19 (0.5) | 7 (5.8) | 4 (5.1) | <0.001 |
| Anosmia and tiredness or myalgia | 180 (4.3) | 123 (3.1) | 11 (9.2) | 46 (58.2) | <0.001 |

Data are median n (%). P-values comparing dentists COVID-19 Test status (no test, negative or positive) are from Fisher’s exact test.

**Table S4. Clinical phenotypes associated with COVID-19 among dentists**
