# Supplementary material for: Prevalence and risk indicators of first-wave COVID-19 among oral health-care workers: A French epidemiological survey
Source: PLoS One. 2021 Feb 11;16(2):e0246586. doi: 10.1371/journal.pone.0246586 (PMC7877573; doi:10.1371/journal.pone.0246586)
Supplement: S5 Table — (DOCX) [file pone.0246586.s005.docx]

|  | **Symptomatic dental assistants (n=360)** | **Symptomatic dentists (n=1097)** | **p-value** |
| --- | --- | --- | --- |
| **Symptoms** |  |  |  |
| Types |  |  |  |
| Fever (>38°C) | **140 (38.9)** | **350 (31.9)** | **0.018** |
| Chills | 116 (32.2) | 372 (33.9) | 0.6 |
| Headache | 7 (1.9) | 27 (2.5) | 0.717 |
| Conjunctivitis | 43 (11.9) | 128 (11.7) | 0.963 |
| Tiredness | 267 (74.2) | 796 (72.6) | 0.598 |
| Rhinitis | **123 (34.2)** | **441 (40.2)** | **0.048** |
| Myalgia | 182 (50.6) | 567 (51.7) | 0.755 |
| Sore throat | **236 (65.6)** | **589 (53.7)** | **<0.001** |
| Cough | 248 (68.9) | 700 (63.8) | 0.091 |
| Anosmia | **32 (8.9)** | **205 (18.7)** | **<0.001** |
| Agueusia | **33 (9.2)** | **180 (16.4)** | **0.001** |
| Difficulty in breathing | **50 (13.9)** | **261 (23.8)** | **<0.001** |
| ARDS | **23 (6.4)** | **26 (2.4)** | **<0.001** |
| Date symptoms first appeared | 2020-03-15 [2020-03-04, 2020-03-22] | 2020-03-14 [2020-03-05, 2020-03-20] | 0.067^*^ |
| **Contact history** |  |  |  |
| Private sphere | **66 (18.3)** | **130 (11.9)** | **0.002** |
| Spouse | 18 (5.0) | 36 (3.3) | 0.181 |
| Child | **25 (6.9)** | **45 (4.1)** | **0.041** |
| Maid | 0 (0.0) | 1 (0.1) | 1 |
| Medical appointment | **8 (2.2)** | **2 (0.2)** | **<0.001** |
| During public transportation | **19 (5.3)** | **25 (2.3)** | **0.007** |
| During travel | 9 (2.5) | 53 (4.8) | 0.08 |
| Unknown | **37 (10.3)** | **49 (4.5)** | **<0.001** |
| Work environment | **206 (81.4)** | **373 (45.9)** | **<0.001** |
| Patient |  |  | **0.022** |
| No suspicion | 214 (84.6) | 726 (89.4) |  |
| Symptoms | 27 (10.7) | 71 (8.7) |  |
| Positive COVID status | 12 (4.7) | 15 (1.8) |  |
| Coworker | **62 (17.2)** | **49 (4.5)** | **<0.001** |
| Assistant | 1 (0.3) | 3 (0.3) | 1 |
| Secretary | **38 (10.6)** | **38 (3.5)** | **<0.001** |
| Nurse | **10 (2.8)** | **6 (0.5)** | **0.001** |
| Unknown | **116 (32.2)** | **245 (22.3)** | **<0.001** |
| **Professional exposure** |  |  |  |
| Types of dental care |  |  |  |
| Clinical interview | 0 (0.0) | 6 (0.5) | 0.351 |
| Dental procedures | **247 (68.6)** | **545 (49.7)** | **<0.001** |
| Number of treated patients |  |  | **<0.001** |
| <10 | 15 (6.0) | 47 (5.8) |  |
| 10 – 50 | 56 (22.5) | 170 (20.9) |  |
| 50 – 100 | 62 (24.9) | 313 (38.5) |  |
| 100 – 150 | 48 (19.3) | 172 (21.1) |  |
| 150 – 200 | 33 (13.3) | 73 (9.0) |  |
| >200 | 35 (14.1) | 39 (4.8) |  |
| Proportion of treated children < 5 years old |  |  | 0.673 |
| 0% | 77 (43.3) | 266 (48.4) |  |
| <25% | 90 (50.6) | 258 (46.9) |  |
| 25% – 50% | 9 (5.1) | 19 (3.5) |  |
| 50% – 75% | 2 (1.1) | 6 (1.1) |  |
| 100% | 0 (0.0) | 1 (0.2) |  |
| Proportion of treated children > 5 years old |  |  | **0.001** |
| 0% | 25 (15.2) | 81 (14.7) |  |
| <25% | 88 (53.7) | 305 (55.4) |  |
| 25% – 50% | 19 (11.6) | 42 (7.6) |  |
| 50% – 75% | 32 (19.5) | 79 (14.3) |  |
| 100% | 0 (0.0) | 44 (8.0) |  |
| PPE |  |  |  |
| No specific measures | 249 (69.2) | 812 (74.0) | 0.084 |
| FFP2 mask | **14 (3.9)** | **96 (8.8)** | **0.004** |
| Safety goggles | **141 (39.2)** | **680 (62.0)** | **<0.001** |
| Hairnets | **26 (7.2)** | **133 (12.1)** | **0.013** |
| Shoe covers | 5 (1.4) | 22 (2.0) | 0.598 |
| Disposable gown | 17 (4.7) | 62 (5.7) | 0.588 |

Data are median [IQR], n (%). p values comparing symptomatic dental assistants and symptomatic dentists are from Fisher’s exact test. ARDS: acute respiratory distress syndrome; PPE: personal protective equipment. Regarding professional exposure, respondents were asked to define types of dental care, number and age of treated patients and PPE used in the 15 days preceding the onset of symptoms.

**Table S5. Putative exposure history in all symptomatic respondents**
