# Supplementary figures and images for: Prevalence and risk indicators of first-wave COVID-19 among oral health-care workers: A French epidemiological survey
Source: PLoS One. 2021 Feb 11;16(2):e0246586. doi: 10.1371/journal.pone.0246586 (PMC7877573; doi:10.1371/journal.pone.0246586)

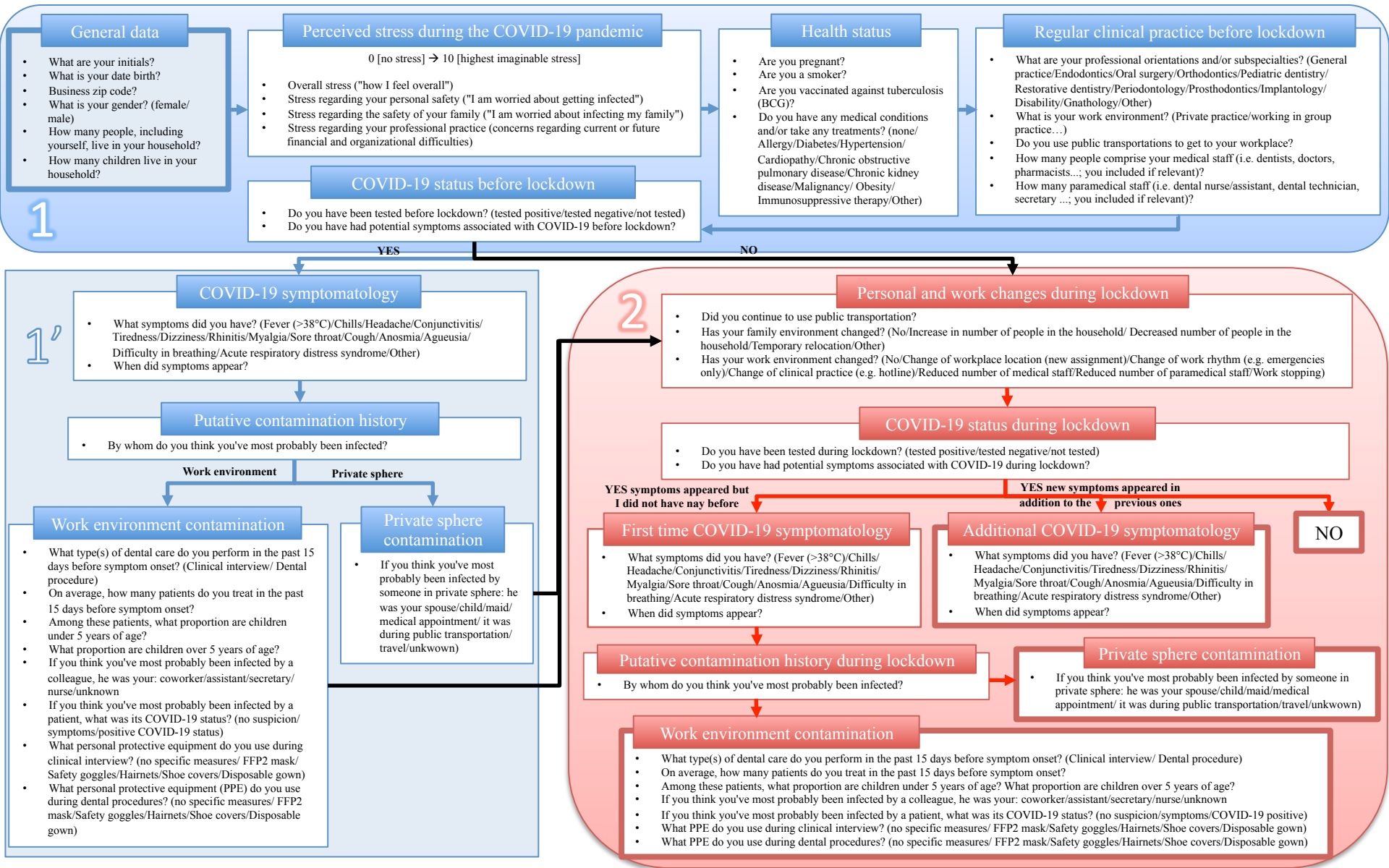

Supplement: S1 Fig — (PDF) [file pone.0246586.s006.pdf]
